# The feasibility, acceptability and preliminary testing of a novel, low-tech intervention to improve pre-hospital data recording for pre-alert and handover to the Emergency Department

**DOI:** 10.1186/s12873-018-0168-3

**Published:** 2018-06-25

**Authors:** David Fitzpatrick, Douglas Maxwell, Alan Craigie

**Affiliations:** 1 Faculty of Health Sciences and Sport, University of Stirling, Stirling, FK9 4LA Scotland; 2 Royal Alexandra Hospital Paisley, NHS Greater Glasgow and Clyde, Corsebar Rd, Paisley, Glasgow, PA2 9PN Scotland; 3 Scottish Ambulance Service , 28 Laverockhall St, G21 4AB, Glasgow, Scotland

**Keywords:** Paramedic, Ambulance, Emergency department, Handover, Pre-alert, Intervention, Feasibility

## Abstract

**Background:**

Poor communication during patient handover is recognised internationally as a root cause of a significant proportion of preventable deaths. Data used in handover is not always easily recorded using ambulance based tablets, particularly in time-critical cases. Paramedics have therefore developed pragmatic workarounds (writing on gloves or scrap paper) to record these data. However, such practices can conflict with policy, data recorded can be variable, easily lost and negatively impact on handover quality.

**Methods:**

This study aimed to measure the feasibility and acceptability of a novel, low tech intervention, designed to support clinical information recording and delivery during pre-alert and handover within the pre-hospital and ED setting. A simple pre and post-test design was used with a historical control. Eligible participants included all ambulance clinicians based at one large city Ambulance Station (*n* = 69) and all nursing and physician staff (*n* = 99) based in a city Emergency Department.

**Results:**

Twenty five (36%) ambulance clinicians responded to the follow-up survey. Most felt both the pre-alert and handover components of the card were either ‘useful-very useful’ (*n* = 23 (92%); and *n* = 18 (72%) respectively. Nineteen (76%) used the card to record clinical information and almost all (n = 23 (92%) felt it ‘*useful*’ to ‘*very useful*’ in supporting pre-alert. Similarly, 65% (*n* = 16) stated they ‘*often*’ or ‘*always*’ used the card to support handover. For pre-alert information there were improvements in the provision of 8/11 (72.7%) clinical variables. ​ Results from the post-test survey measuring ED staff (*n* = 37) perceptions of handover demonstrated small (*p* < 0.05) improvements in handover in 3/5 domains measured.

**Conclusion:**

This novel low-tech intervention was highly acceptable to ambulance clinician participants, improving their data recording and information exchange processes. However, further well conducted studies are required to test the impact of this intervention on information exchange during pre-alert and handover.

**Electronic supplementary material:**

The online version of this article (10.1186/s12873-018-0168-3) contains supplementary material, which is available to authorized users.

## Background

Poor communication during patient handover is recognised internationally as a root cause of a significant proportion of preventable deaths [1] and as such is named as one the of the top five World Health Organisation improvement priorities [2]. Despite the introduction of a number of recommended strategies to reduce this harm, the handover phase of care continues to impact negatively on patient outcome [3–6]. It is widely accepted that handover of patients in any clinical setting carries significant risk [7]. However, handover of critically ill or injured patients from the pre-hospital to Emergency Department (ED) team carries additional risk due to the time critical nature of the process and multiple human factors involved in dealing with stressful clinical events [5, 8]. Such knowledge has led to calls for further, more rigorous research into handover, and to develop solutions to support handover in diverse environments [5, 9, 10].

Despite the limited evidence on pre-hospital to ED handover practice, a number of pragmatic options, informed by theory, have been recommended. These include for example, the development of shared mental models, standardisation of approach (an agreed handover system and format/mnemonic) applied at the interface between professional domains, and the introduction of technology to support and enhance the process [5, 7, 11]. But unfortunately, cultivating a movement towards a shared mental model has remained a challenge with evidence of considerable inconsistencies in mnemonic usage and preference both nationally and internationally [12–15]. Beyond these higher level, theoretically informed systems and processes, there are other more fundamental and pragmatic challenges to consider and address.

Recording clinical data used during handover accurately and with ease is incredibly challenging during the delivery of prehospital care. These data are the cornerstone of clinical handover, independently or aggregated, they provide an immediate clinical impression of the patient’s condition [16, 17]. Excluding some of these essential variables during pre-alert or handover may delay subsequent life-saving interventions [18, 19]. The importance of developing tools to support the recording and handover of these data is established in the study by Bhabra et al. [20] who identified only 33% of data is retained on first handover when relying on memory alone, but where a standardized, printed form was used, 99% of information was retained. Rightly, therefore, attention has recently been directed towards the use of technology in supporting both data capture and handover with the development of natural language generation software [21, 22] and in exploring ambulance clinicians use of electronic patient report forms [23]. Hardware placed in ambulances to support the recording of clinical data used during handover is often in tablet form. However, this technology is not always carried by ambulance clinicians to the patient’s side, particularly in those incidents that are time-critical. Consequently, data is often retrospectively entered, post-handover. And whilst technology continues to advance in this area, more rapid and accessible methods are required now to support the recording of essential clinical data in the high acuity patient.

Pragmatic workarounds have been developed by ambulance clinicians and currently it is not uncommon for clinical data to be recorded on the back of a gloved hand or on scrap paper [21, 24, 25]. However, there are potential infection control risks with the former and neither practice necessarily comply with data protection legislation. Additionally, data recorded will be variable and with heavy contamination of gloves being a particular problem in these environments, glove changes can be frequent [26]. As such, both gloves or paper will be easily discarded or lost.

There currently exists a paucity of evidence, and therefore understanding and knowledge, on how ambulance clinicians can best record and deliver information essential for handover. There is therefore an immediate need to investigate and develop more pragmatic and low-tech solutions to record information that supports information exchange between ambulance clinicians and ED staff.

## Methods

This project aimed to measure the feasibility and acceptability of a novel intervention designed to support clinical information recording and delivery during pre-alert and handover within the pre-hospital and ED setting.

### Design and setting

A simple pre and post-test design was used with a historical control. The Scottish Ambulance Service (SAS) is a national service covering 30,420 sqm, serving a population of 5.4 million [27] and responding to around 650,000 emergency calls per annum [28]. The service is primarily set within an Anglo-American model of care [29] whereby road based Paramedics and Emergency Medical Technicians (EMT) deliver the majority of care. At the time of study the service operated within 5 geographical Divisions (North, East, South, West and West Central). The study was undertaken in West Central Division, which covers the most densely populated areas in the West of Scotland. Our intervention hospital and ambulance station were therefore located in a busy urban setting in the centre of Glasgow; population 606,000. During the 3 month study period (February to April 2017) the ED received 22,913 patients [30], with Ambulance Clinicians at the intervention station responding to circa 5339 emergency calls during the same time period [31].

### Participants

Eligible participants included all ambulance clinicians (*n* = 69) based at one city centre Ambulance Station and all nursing and physician staff (*n* = 99) based in the ED of a large city hospital in Glasgow. The ambulance station selected was the largest in Glasgow and, as such, provided a broad demographic pool of clinicians; clinical grade EMT’s 45% [*n* = 31] vs. Paramedic 55% [*n* = 38]) and clinical experience (service in years range from 1 to 40). Due to organisational time pressures we intentionally kept to a minimum the data requested from ED staff and so only demographic data on job title was sought (Nurse/Physician). All ED participants were invited to complete the respective pre and post-test questionnaires.

### Intervention design

The intervention design was informed by the collective results of two unpublished studies i) a survey investigating handover in the pre-hospital domain and ii) a pre-test survey of handover practices of local ambulance clinicians (*n* = 23/69; 33%). A study stakeholder group was formed to synthesize the results of both along with published evidence and particularly within the context of existing challenges affecting key points in the handover process: pre-handover, arrival, handover and post-handover [7]. A specific focus was retained on the technical aspects of information recording in both the prehospital and in-hospital settings. The stakeholder group consisted of front line ambulance clinicians (*n* = 6), an Emergency Medicine physician and ED registrar, senior paramedic managers (*n* = 4), an applied health service researcher and a Union representative. Infection Control and Data Protection Leads from the SAS ensured compliance with existing legislation and authorised the final prototype used in testing.

A graphic design expert designed the card based on the stakeholder group’s requirements. The prototype intervention consisted of a double-sided A6 card in high contrast colour with pre-alert and handover clinical information requirements on opposing sides. Corresponding boxes for clinical variables were available for writing in with a marker pen. Included variables and final mnemonic choice were based on the synthesized data and a compromise on both the needs of the ambulance clinicians and ED staff. The variables included were known prognostic indicators (individually or collectively) for degrees of severity in injury and illness [32, 33].

The intervention was piloted in a simulated setting with prehospital critical care practitioners and physicians prior to being finalised. The simulation led to a change in physical format from a card encased in a sealable plastic cover to a stand-alone, high density plastic card (Additional file 1). A number of pens were tested and a type selected that ensured rapid dry and non-smudge properties. To support the concept of a shared mental model, an A4 ‘ambulance form’ (AF) was developed for use within the ED (Additional file 2). This consisted of a pre-printed A4 sheet placed next to the ambulance radio located within the ED; this is the radio system that receives pre-alert calls from ambulance clinicians who are bringing in critically ill patients. The AF content duplicated the information provided on the ambulance pre-alert and handover card.

### Intervention procedure

Ambulance clinicians (*n* = 69) at the participating station were each issued a personal issue pre - alert and handover card (PAHC) with pens. An instruction notice was placed at various strategic locations around the ambulance station. Cards were to be used in time-critical cases with both the pre-alert and handover sections to be completed contemporaneously with assessment at appropriate times during care. During pre-alert and handover clinical information would be delivered in the sequence provided on the PAHC. For data protection purposes recording of date of birth and surname was not permitted. Post incident, these data were transferred onto the electronic patient report form in the ambulance as per usual practice. In accordance with infection control procedures the cards were cleaned using Tuffie© wipes and dried with a paper towel.

Within the ED, those receiving the ambulance pre-alert were instructed to record all clinical information provided via the radio call in the respective boxes on the AF. On arrival within the ED ambulance clinicians then verbally delivered the clinical information in the sequence provided on PAHC. Receiving staff would record this handover information in the respective sections on their AF.

### Data collection tools

#### Pre-test measures

##### Subjective

During February 2016 opinions of ED staff on ambulance clinician handover were measured via a short online survey (Additional file 3). In the absence of rigorously validated and accepted measures of perceptions of pre-alert and handover quality in the ED, the survey development was informed through key elements of handover identified in the study by Ledema et al. [3] along with the clinical experiences of study investigators. Due to the significant pressures of the ED environment, the survey was deliberately designed to be short and succinct and thus reduce time required for completion. Five domains of handover were measured using a five point Likert scale (1- Always to 5 – Never). The domains measured were: i) Structure ii) standardised iii) Focus iv) repetition avoidance and v) interruption avoidance. Potential participants (*n* = 99) received an e-mail containing a link to the online survey along with information on the project. Consent was presumed by completion of the questionnaire. These data formed the historic control.

##### Objective

Base-line data on pre-alert and handover were extracted from the month preceding the intervention introduction (January 2017) using the paper-based AF which had already been introduced for use by ED staff. Key clinical data were recorded on these sheets during the pre-alert and handover. Ambulance Clinicians in the intervention station were not made aware of the study prior to its commencement.

#### Post-test measures

##### Subjective

Following the 3 month intervention test period (February – April 2017) questionnaires were sent to both ED and Ambulance Clinicians during May 2017. Ambulance clinicians were sent a paper-based questionnaire (Additional file 4), while ED staff were invited again, via e-mail, to complete the online questionnaire (Additional file 3). The different questionnaire formats reflected the operational environments for each clinical group and were designed to improve response rates. Ambulance Clinicians are established as hard to reach populations in questionnaire based research and traditionally deliver low response rates [34–36]. The decision to use paper-based surveys for ambulance clinicians was informed by the predictable difficulties experienced with a highly mobile workforce, their inability to access computers and past studies undertaken in similar settings [37, 38] that used evidence informed methods [39, 40] which had previously provided improved response rates. The questionnaire for ambulance clinicians focused on measuring the feasibility and acceptability of the prompt card. Questions consisted of Likert scales and dichotomised yes/no responses. ED staff repeated the same questions used in the pre-test questionnaire. This permitted comparisons between the pre-test (historical control) and post-test measures.

Prior to distribution, questionnaires were assessed for ease of completion and comprehension. This led to a small number of changes in wording to aid comprehension. Again participants were provided with a cover letter and information sheet with consent presumed by completion of the questionnaire. For ambulance clinician participants pens were provided in the invitation envelops and a cardboard ‘post box’ located in the ambulance station mess room to facilitate ease of completion and submission.

##### Objective

Ambulance Forms were routinely completed by ED Nurses during handover and collected by receptionists thereafter. These forms were divided into i) a pre-alert information section and ii) a handover information section and included the same variables as were presented in the ambulance clinician’s pre-alert and handover cards. Frequency counts were used to measure clinical variables recorded during both the pre-alert and handovers provided by ambulance clinicians. These data permitted pre and post-test data comparisons to be made on the interventions impact.

### Analysis

Data were recorded on Microsoft Excel 2010 prior to conversion to Statistical Package for the Social Sciences (SPSS) (v.21). Descriptive statistics were used to present the results of the questionnaire. Chi Square tests were used to compare the frequency counts between the hospital pre-alert pre and post-test objective data. Mann Whitney U analysis was used to compare the median ratings of the pre and post-test measures on opinions of handover quality.

## Results

### Ambulance participants

A four week intervention run-in period from 1st to 28th February was permitted to enable ambulance clinicians and ED staff to familiarise with the intervention. During the 3 month study period ambulance clinicians at the intervention station responded to 5339 call and conveyed 1938 (30%) of these patients to the participating ED. There were approximately 160 (20/week) pre-alerts made to the ED during this time period. Thirty six percent of ambulance clinicians (*n* = 25) completed the post-test questionnaire; paramedics 70.8% (*n* = 18) vs Technicians 29.2% (*n* = 7). The median length of service in years was 16 (IQR 8–30).

### Acceptability and perceived utility of intervention

Ninety six percent (*n* = 24) of participants reported receiving the intervention. Most felt both the pre-alert and handover components of the card were ‘useful’ to ‘very useful’ (92%; *n* = 23 for pre-alert and 72%; *n* = 18) for handover). In measuring the utility of the intervention, questions focused on the use of the card, its legibility, format and pen performance. Over three quarters of responders (76%; *n* = 19) reported using the card to record clinical information and almost all (92%; *n* = 23) felt it ‘*useful*’ to ‘*very useful*’ in supporting pre-alert. A small proportion 16% (*n* = 4) reported recording information on back of gloved hand and 8% (*n* = 2) still relying on memory. Almost two thirds of participants (65%; *n* = 16) stated they ‘often’ or ‘always’ used the card for handover.

The pen was perceived to perform well with 60% rating it either ‘good’ (12%; *n* = 3) or ‘very good’ (48%; *n* = 12). However, a small proportion (12%; *n* = 3) rated the pen as either ‘poor’ or ‘very poor’. Perceptions on the cards legibility in both bright or poor lighting were also sought. Sixty four percent (*n* = 16) rated it as ‘easy’ (24%; *n* = 6) or ‘very easy’ (40%; *n* = 10) to read in bright lighting and 56% (n = 16) ‘easy’ (24%; n = 6) or ‘very easy’ (32%; *n* = 8) to read in poor lighting. Forty percent (n = 10) were neutral in their response.

### Emergency department participants

Thirty seven percent (*n* = 37/99) of ED Staff responded to the pre-test questionnaire; Doctors 63.2% (*n* = 24) and Nurses 36.8% (*n* = 13). Slightly less responded to the post-test questionnaire; 29% (*n* = 29/99); Doctors 48% (*n* = 14) and Nurses 52% (*n* = 15).

### Perceptions of handover quality

ED staff (Nurses and Physicians) were asked to rate their experiences of different aspects of Ambulance Clinician handover (as per ‘domains’ outlined in methods section previously). The overall responses in the pre-test survey were relatively neutral but clearly demonstrated a requirement for improvement in each of the handover domains measured. Results from the post-test survey demonstrated small but statistically significant improvements in perceptions of handover in 3/5 domains measured (Table 1).

**Table 1:** Comparing ED clinicians pre-test and post-test perceptions of handover (Likert scale – 1 = always to 5 = never). Mann-Whitney *U* test comparing median responses

Aspect of handover measured	Pre-test survey results	Post-test survey results	*p*-value (U)
	Median (IQR^a^)	Median (IQR^a^)	
Is structured	3 (2–3)	2 (2–3)	0.126
Is standardised	4 (2–4)	2 (2–3)	<.001
Is focussed	2 (2–3)	2 (2–2.75)	0.223
Avoids repetition	3 (2–3)	2 (2–2.75)	0.014
Avoids interruptions	3 (2.25–4)	2 (2–3)	0.003

^a^*IQR* Inter-quartile range

### Objective measure of pre-alert information

**Fig. 1:**
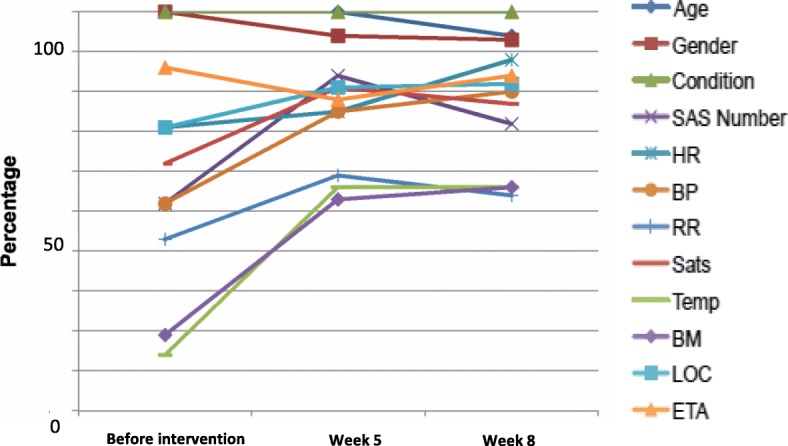
Trends in clinical variables provided during pre-alert

Ambulance pre-alert forms were routinely used within the ED throughout both the pre-and post-test stages of the study. To provide an indication of any change in handover clinical information provision data were taken from three time points i) the month leading up to the intervention introduction, ii) the end of week 5 (after the 4 week bedding in period) and iii) at week 8. Figure 1 demonstrates the trend in variables provided during pre-alert starting at beginning of week 5 of intervention period. Frequency counts and proportions of the variables recorded during pre-alert are presented in Table 2. There was a statistically significant improvement in the delivery of 2/7 (29%) clinical variables during pre-alert. However, non-significant improvements were apparent in the remaining five other clinical variables. Small, non-significant reductions, were also noted in three other variables. Overall demonstrable improvements were identified in 8/12 (67%) variables.

**Table 2:** Recorded frequencies of pre-alert variables provided to ED pre and post-test

Pre-alert variable	Pre-test forms % (*n* = 21)	Post-test forms % (*n* = 32)	% change	*p*-value chi square
Call sign	52.4% (*n* = 11)	71.9% (*n* = 41)	+19.5	.379
Age	100% (*n* = 21)	100.0% (*n* = 32)	n/a	n/a
Gender	100% (*n* = 21)	93.8% (*n* = 30)	- 6.2	.243
Injury/Illness	100% (*n* = 21)	100% (*n* =32)	n/a	n/a
LOC^a^	71.4% (*n* = 15)	83.9% (*n* = 26)	+12.5	.281
Resps^a^	42.9% (*n* = 9)	59.3% (*n* = 19)	+16.4	.239
Sats^a^	61.9% (*n* = 13)	81.3% (*n* = 26)	+19.4	.118
Heart rate^a^	71.4% (*n* = 15)	75% (*n* = 24)	+3.6	.773
Blood pressure^a^	52.4% (*n* = 11)	75% (*n* = 24)	+22.6	.089
Blood Glucose^a^	19% (*n* = 4)	53.1% (*n* = 17)	+34.1	.013*
Temp^a^	14.3% (*n* = 3)	56.3% (*n* = 18)	+42	.002*
ETA	85.7% (*n* = 18)	78.1% (*n* = 25)	-7.6	.490

^a^Clinical variables only

*Chi square analysis with an alpha level of 0.05 (exact Sig. [2-sided])

Analysis of the handover section of the ambulance forms within the ED proved problematic. Of the forms that were collected only 32/57 (56%) had at least one of the data fields completed. Only 10.5% (*n* = 6) were fully completed and as a result comparisons with pre-test data were not undertaken. And so, whilst the results suggest a perceived improvement in the quality of handover this could not be measured more objectively as had originally been planned.

## Discussion

We aimed to test the feasibility and acceptability of a novel low tech intervention to support the prehospital recording and delivery of clinical information for use by ambulance clinicians during pre-alert and handover. Our measures focused on ambulance clinician’s perceptions of the intervention and both subjective and objective elements of the pre-alert and handover process from the ED clinician’s perspectives. We also sought to determine whether the data collection tools developed for this study would be sufficient in providing useful measures of improvement in a larger trial.

### Acceptability of the intervention

Our study has determined that, overall, Ambulance Clinician participants felt the intervention was useful to very useful, using it to both record and support the delivery of clinical information in the majority of pre-alert and handovers. The perceptions of participating ED staff on handover quality were also positive with significant improvements in 3 out of 5 handover domains measured and small, non-significant improvements in the remaining 2 domains. During pre-alert, despite statistically significant improvements in the provision/recording of only *n* = 2 (29%) clinical variables, overall, positive trends were identified in *n* = 8 (67%) variables. Had we extended final measures beyond the 12 weeks, permitting more familiarity with the intervention and embedding in clinical practice, these figures may have continued to improve.

### Feasibility of the introduction of the low-tech intervention

Our intervention was designed to be intuitive, practical and one that, with minor modification, would seamlessly replace existing clinical data recording practices when managing high acuity patients. As such we decided that no formal education would be provided to ambulance clinicians to support the interventions introduction (with the exception of station notices). There were possible risks associated with this approach however. Educational practices within the host ambulance service frequently consist of clinical bulletins posted in station boards or distributed via e-mail and intranet. The efficacy of this educational format has not been formally measured in this setting, however some research has established that similar ‘online’ forms of education for ambulance clinicians does not necessarily improve adherence to guidance [24]. Additionally, other studies strongly advocate education and training in handover ([25, 41]. The finding that this deliberately pragmatic and considered approach was well accepted by participating clinicians, and that so many ambulance clinicians reported using the intervention for its intended purpose were therefore welcome. This also may suggest that this intuitive intervention could be introduced on a larger scale in future trials with little requirements for formal education.

### Initial assessment of the interventions ability to share clinical data

Although this study was not designed or powered to address the question of intervention efficacy, we accessed the data recorded on the AF’s within the ED to identify trends. Previous work using similar measures [42] determined that an average 50% of clinical variables were received from ambulance clinicians during handover, but that only 72.9% of the information received was documented by ED staff. Although we only report on pre-alert rather than handover data, our pre-intervention average for vital signs provided/recorded was 47.6%, slightly lower than those reported by Carter et al. [42]. However, post intervention, our proportion of clinical variables provided/recorded improved to an average of 69.4%; a 46% increase and proportionately higher than in the study by Carter et al. [42]. Although we were unable to objectively determine if these improvements were as a direct result of the intervention, no other changes in pre-alert or handover were introduced during the study period. And so these identified difference are likely to be a result of the intervention and provide an indication that it may have positively impacted on data provision during pre-alert.

It should be noted too that we are also comparing different stages and forms of communication to those presented in the study by Carter et al. [42] i.e. pre-alert via radio call v’s face-to-face handover. There are distractions and pressures likely to present during a face-to-face handover in the ED that do not exist during radio communication; the patient for one. These are likely to impact on the amount and quality of data provided and recorded during handover and may therefore negatively impact on the proportions of variables recorded [25, 41]. Our study does however highlight the importance of this pre-alert phase of information exchange prior to the physical handover. The ability to provide timely and accurate clinical information prior to arrival, and handover, at the ED has been shown to improve care in specific time critical conditions [43, 44]. It is advisable therefore that such pre-arrival information is considered as an essential and central component of the broader handover process and system.

Our measured indications of improvements may also have been influenced by the introduction of a common system for use by both ambulance and ED clinicians. We fostered the shared mental model concept [7] by ensuring both professional groups used the same mnemonic and as such a common understanding of clinical variables required and provided was developed within the system. Ensuring a common expectation on the provision of specific clinical variables has also been shown to improve efficacy of handover [45]. The ability to manually and accurately record pre-alert information contemporaneously [20] negates the requirement for information retention during periods of high cognitive demand and also appears to have supported this process. Either these individual components, or their cumulative parts, may have contributed to the improvement in perceptions of ED participants and the proportion of clinical data provided or recorded during pre-alert.

Notably there were no statistically significant improvements in two of the subjective handover domains measured; ‘*structured*’ and ‘*focused*’. On reviewing the AF format it was clear that the sequence of clinical data boxes, particularly in the pre-alert section, did not match the sequence provided on the ambulance PAHC. It is highly probable that this had an impact on the flow of pre-alert and handover recording by ED staff and may partially explain why there were no perceived improvements in these variables measured. This was valuable learning and as such changes will be incorporated into future interventions to ensure continuity of clinical variable sequence in both the PAHC and AF.

### Challenges and considerations for recording tools aimed at measuring pre-alert and handover data

ED staff consistently used the ambulance pre-alert section of the form to record the pre-alert information thus suggesting this section of the form was a useful and feasible measurement tool. It should be noted that there are likely to be occasions whereby ambulance clinicians, working alone in the rear cab of an ambulance, will be required to prioritise vital signs measured. For example, during management of an unconscious patient the absolute need to provide airway management will take priority over a temperature recording. Similarly the check and identified presence of a radial pulse in a time critical patient serves as a proxy measure of blood pressure and may replace, at least in the short term, the need to record a blood pressure. It is likely therefore that these practical elements may partly explain the variability in frequency counts and inability to consistently reach 100% of clinical variables provided during pre-alert. It also highlights the difficulties in ensuring that all clinical variables are ‘always’ provided during pre-alert and handover. Occasionally, this is not feasible and should be considered as a limitation to any objective measure.

However, problems were encountered with the completion of the handover section. There were two elements that may have influenced this. Firstly, in our ED site handover data from ambulance clinicians were not always recorded on the AF contemporaneously. This is not unusual in the ED setting. Well known pressures on ED’s i.e. crowding and capacity issues lead to distractions known to impact on the accuracy of information recording [41], and perhaps on a reliance on recall of verbal information, previously identified as a contributor to information loss [20, 41, 46]. Secondly, and perhaps a more significant contributor to data loss, were our data collection methods. To ensure data capture, clerical staff were instructed to photocopy the AF’s at (or very near to) the time of handover. Ironically this may have led to forms being removed too early and before full completion. Thus, the investigators enthusiasm to capture data early is likely to have had a significant impact on the recording of handover data. These combined factors, therefore, are probable contributors to the loss of information on AF’s. Essentially, future studies must ensure rigorous and timely methods of data recording to support the objective measure of the handover component of new interventions. Consideration of an observational component via video recording [47] or an external, supernumerary, observer for contemporaneous note taking would also be helpful and advisable in future studies.

These identified issues also highlight the complex nature of the handover process and the need for investigation beyond just the technical aspects [7]. Low tech or high tech interventions may provide solutions to support the recording and delivery of known essential clinical information during handover, however, more research is clearly required to investigate the non-technical and system elements of ambulance clinician to ED handover. Such systems will undoubtedly require multi-modal interventions to ensure the accurate transfer of clinical information during high acuity situations. As such, our novel low-tech intervention tested in this small-scale study may perhaps be viewed upon as one small component of a larger system of handover. A system that needs further development of multiple components in order to address both the technical and non-technical aspects of handover. Only when these multiple elements of handover are addressed will any true improvements in handover quality and safety be realised.

#### Strengths and limitation

This was a small-scale study to measure utility and acceptability of a novel intervention. It was conducted in one large city centre ambulance station in Scotland. However, the results were positive and demonstrated the ease at which a simple, pragmatic intervention could be introduced within the prehospital domain. The ambulance clinician response rate was low (36%, *n* = 25) and, as such, is prone to response bias. However, in terms of demographics we found no difference in median length of service between study participants (*Mdn* = 16; IQR 8–30) and overall station population (*Mdn* = 15; IQR 3.5–23.75); *U* = 715; *p* = 0.247. Similarly, there were no difference between proportions of grade; paramedics 55% (*n* = 38) vs 70.8% (*n* = 18) and Technicians 45% (*n* = 31) vs 29.2% (*n* = 7); χ^2^ (1, *N* = 94) = 1.83, *p* = 0.176. Despite these similarities in demographics, low response rates and small sample size limit the results generalisability to the wider ambulance population and as such these results should be interpreted with caution. However, the study was not designed to be generalisable or provide a definitive answer to the interventions effectiveness on improving handover. The ‘Hawthorne effect’ may also have been present during this study with a positive impact on ambulance clinicians pre-alert and handover behaviours [48]. Further research is planned on a larger scale across one Division in Scotland.

The key learning that has taken place during the undertaking of this study will support improvements to methods used to further test both the subjective and objective efficacy of the intervention. Specifically these will include the introduction of additional evidence based methods to improve response rates to questionnaires with consideration also to the addition of online access (via our cab-based computer systems), the need for qualitative components by way of interviews and the use of video footage or observers to objectively record elements of handover.

## Conclusion

These results suggest that the introduction of a low tech, novel intervention is very acceptable to ambulance clinicians, intuitive to use, requires little education on use and positively supports their contemporaneous data recording and information exchange processes. Although there are some data to suggest the effectiveness of the intervention in its ability to improve information sharing during pre- alert and handover, this study was neither designed nor powered to do this. However, the overall positive results suggest that further well conducted studies to test an updated intervention, based on feedback provided, are worthwhile. Importantly, future studies should carefully consider, in the absence of advanced data sharing technology, how information required for pre-alert and handover can be recorded contemporaneously to minimise data loss. This will be essential from both a patient safety perspective and to more objectively measure the impact of any future handover intervention.

## Additional files

Additional file 1: Pre-alert and Handover Card (PAHC). This is the plastic, double sided, pre-alert and handover card issued to Ambulance Clinicians. (PDF 84 kb)

Additional file 2: Ambulance Form (AF). This is the paper based Ambulance Form used by Emergency Department staff to record the ambulance clinicians pre-alert and handover information. (PDF 71 kb)

Additional file 3: Emergency Department Handover Questionnaire. These are the questions used in the online questionnaire to measure ED staff perceptions of ambulance clinician handover. (PDF 20 kb)

Additional file 4: Ambulance Questionnaire. This the paper based questionnaire used to measure ambulance clinicians perceptions of the feasibility and acceptability of the prompt card. (PDF 179 kb)

## Abbreviations

AF: Ambulance Form
ED: Emergency Department
EMT: Emergency Medical Technician
PAHC: Pre-alert and Handover Card
SAS: Scottish Ambulance Service
