# Supplementary material for: The feasibility, acceptability and preliminary testing of a novel, low-tech intervention to improve pre-hospital data recording for pre-alert and handover to the Emergency Department
Source: BMC Emerg Med. 2018 Jun 25;18:16. doi: 10.1186/s12873-018-0168-3 (PMC6019792; doi:10.1186/s12873-018-0168-3)
Supplement: Supplementary file 1 — Pre-alert and Handover Card (PAHC). This is the plastic, double sided, pre-alert and handover card issued to Ambulance Clinicians. (PDF 84 kb) [file 12873_2018_168_MOESM1_ESM.pdf]

Scottish Ambulance Service

Pre-alert and Handover aide-memoire

NHS SCOTLAND

Pre-alert

Age ____ years Gender M ☐ F ☐

Injury/Illness ____

GCS	
RR	
SpO2	
HR	
BP	/
BM	
Temp	
NEWS	
ETA	

Eye opening
4 Spontaneous
3 To voice
2 To pain
1 Nil

Verbal response
5 Orientated
4 Confused
3 Inappropriate words
2 Incomprehensible sounds
1 Nil

Motor
6 Obeys commands
5 Localises to pain
4 Withdraws from pain
3 Abnormal flexion
2 Abnormal extension
1 Nil

Handover (IMIST)

Scottish Ambulance Service NHS SCOTLAND

Identification

First name ____ Age ____ years

Mechanism/Medical complaint

Injuries/Information related to complaint

Signs/Symptoms

GCS		RR		SpO2		HR	
BP	/	BM		Temp		NEWS	

Key symptoms:

Treatment (ET, IV, Drugs etc.)

Trends (improved/deteriorated)
