# Supplementary material for: The feasibility, acceptability and preliminary testing of a novel, low-tech intervention to improve pre-hospital data recording for pre-alert and handover to the Emergency Department
Source: BMC Emerg Med. 2018 Jun 25;18:16. doi: 10.1186/s12873-018-0168-3 (PMC6019792; doi:10.1186/s12873-018-0168-3)
Supplement: Supplementary file 2 — Ambulance Form (AF). This is the paper based Ambulance Form used by Emergency Department staff to record the ambulance clinicians pre-alert and handover information. (PDF 71 kb) [file 12873_2018_168_MOESM2_ESM.pdf]

STANDBY NOTIFICATION & HANDOVER FORM

Glasgow Royal Infirmary Emergency Department(adapted from IMIST-AMBO)

Patient Label

**Date:** DD/ MM/2015		**Time:**	
**Age:**	**Gender:** M / F	**SAS Number:**	
**Condition:**			☐ Cardiac Arrest<br>☐ Trauma Team

OBSERVATIONS PREHOSPITAL

HR:	BP:	RR:	SaO_2:air/O2	BM:
**Conscious Level**	GCS	ALERT ☐	VERBAL ☐	PAIN ☐ UNCONSCIOUS ☐

INTERVENTIONS PREHOSPITAL

ETT ☐<br>OPA/NPA ☐	IV Access ☐<br>IV Fluids ☐	Oxygen ☐<br>Nebuliser ☐	Other	**ETA:** *Time*

UPON ARRIVAL:

Time:

→*Maintain 20-30 second period where patient remains on stretcher and deliver IMIST information uninterrupted.*

**I**dentification	Confirm Prehospital Details
**M**edical Complaint & **M**echanism	
**I**njuries & Information	
**S**igns& Clinical Findings	
**T**reatment &Trends	

→ ***Transfer over - then QUESTION & CLARIFY where required***

**A**llergies	
**M**edication	
**B**ackground history	Family& If present or following on
**O**ther information	Premorbid / Cognitive / Functional status

→ ***QUESTION & CLARIFY before SAS depart***
