# Supplementary material for: The feasibility, acceptability and preliminary testing of a novel, low-tech intervention to improve pre-hospital data recording for pre-alert and handover to the Emergency Department
Source: BMC Emerg Med. 2018 Jun 25;18:16. doi: 10.1186/s12873-018-0168-3 (PMC6019792; doi:10.1186/s12873-018-0168-3)
Supplement: Supplementary file 3 — Emergency Department Handover Questionnaire. These are the questions used in the online questionnaire to measure ED staff perceptions of ambulance clinician handover. (PDF 20 kb) [file 12873_2018_168_MOESM3_ESM.pdf]

Question: With respect to the following five statements, please rate on the frequency scale provided (1 – always to 5 – never) your perceptions of handover of resuscitation patients:

	Perceived occurrence of domain measured				
Domain measured	Always				Never
Handover is structured	1	2	3	4	5
Handover is standardised	1	2	3	4	5
Handover is focused	1	2	3	4	5
Handover avoids repetition	1	2	3	4	5
Handover avoids interruption	1	2	3	4	5
