# Supplementary material for: The feasibility, acceptability and preliminary testing of a novel, low-tech intervention to improve pre-hospital data recording for pre-alert and handover to the Emergency Department
Source: BMC Emerg Med. 2018 Jun 25;18:16. doi: 10.1186/s12873-018-0168-3 (PMC6019792; doi:10.1186/s12873-018-0168-3)
Supplement: Supplementary file 4 — Ambulance Questionnaire. This the paper based questionnaire used to measure ambulance clinicians perceptions of the feasibility and acceptability of the prompt card. (PDF 179 kb) [file 12873_2018_168_MOESM4_ESM.pdf]

Emergency Department stand-by and handover survey

Please carefully read the following questions about stand-by and handover and place a tick ☑ in the corresponding box or write your answers in the spaces provided (**do not include any patient information**). You do not have to take part in this survey. However, if you do decide to take part, you are free to withdraw at any time. All responses will be completely anonymised. Consent will be presumed by your completion and submission of the survey. **Once completed please fold the survey form and place in the study post box located in the mess room**. If you would like further information about this work please contact Dr David Fitzpatrick at: ***david.fitzpatrick@nhs.net***.

About you

Job Title:

☐ Paramedic

☐ Technician

Number of years on Accident and Emergency duties: ☐

This section is about your STAND-BY (pre-alert) practice

Q1 Are you aware of the services STAND-BY guidance available within the new SAS 'Clinical Guidelines, Pathways and Patient Safety Pocket book'?

☐ Yes (if '**Yes**' please go to **Q2** below)

☐ No (if '**No**' please go straight to **Q3**)

Q2 Do you follow this guidance?

☐ Yes

☐ No (please provide an explanation of why not in space below)

....

....

Q3 With respect to shift 'role' who, most often, will provide the STAND-BY call?

☐ The ambulance clinician driving the ambulance (Driver)

☐ The ambulance clinician in the treatment area of the ambulance (Attendant)

Q4 Which factors can influence who (driver or attendant) provides the STAND-BY? (tick all that apply)

☐ Trying to avoid distressing or worrying patient/relative/escort

☐ The clinician who holds the most information on the patient, whether driver or attendant

☐ Driver too busy; requires to focus on driving

☐ Attendant too busy: patient condition requiring on-going intervention

☐ Confidentiality (ambulance clinicians passing over potentially sensitive information)

☐ Risk to ambulance clinicians i.e. agitated, aggressive or uncooperative patient

☐ Other (please state)...

Please turn over

Q5 How has your current STAND-BY practice been informed? (tick all that apply)

- ☐ Pre-qualification education (Academy/Barony/Redlands)
- ☐ JRCALC National Clinical Guidelines
- ☐ National Clinical Bulletins
- ☐ Service Guidance (SAS Clinical Guidelines, Pathways and Patient Safety Pocket book)
- ☐ Peers/feedback from ED clinicians and colleagues
- ☐ General experience
- ☐ Specific education on Stand-by practice
- ☐ Major Incident Training
- ☐ Learning in Practice (LIP)
- ☐ Other: …………………………………………………………………………………….....................

Q6 Where do you record clinical information used for prehospital STAND-BY? (tick all that apply):

☐ Committed to memory	☐ paperPRF	☐ Back of ungloved hand
☐ Back of gloved hand	☐ ePRF	☐ Notes on scrap paper/notepad

☐ Other:

……
……

Q7 Which of the following elements do you feel must be provided during a STAND-BY call to the ED (tick all that apply, and add in space below if elements not included)

☐ My Call Sign	☐ AVPU	☐ Age
☐ Mechanism of injury	☐ GCS	☐ Gender
☐ Injuries/Illness	☐ Heart rate	☐ Time of insult/injury
☐ Blood Glucose	☐ Respiratory rate	☐ Time on scene
☐ Condition	☐ Systolic BP	☐ Diastolic BP
☐ Change in condition	☐ Temperature	☐ Medical history
☐ Skin Colour	☐ Medication	☐ ETA

Other elements (please add):

……
……

Please turn over

This section is about your HANDOVER practice within the resuscitation room

Q8 Are you aware of the services HANDOVER guidance available within the new SAS 'Clinical Guidelines, Pathways and Patient Safety Pocket book'?

- ☐ Yes (if '**Yes**' please go to **Q9** below)
- ☐ No (if '**No**' please go straight to **Q10**)

Q9 Do you follow this guidance?

- ☐ Yes.
- ☐ No (please provide an explanation of why not in space below)

………
………

Q10 How has your current HANDOVER practice been informed? (tick all that apply)

- ☐ Pre-qualification education (Academy/Barony/Redlands)
- ☐ JRCALC National Clinical Guidelines
- ☐ National Clinical Bulletins
- ☐ Service policy (SAS Clinical Guidelines, Pathways and Patient Safety Pocket book)
- ☐ Peers/feedback from ED clinicians and colleagues
- ☐ Experience
- ☐ Specific education on handover practice
- ☐ Major Incident Training
- ☐ Learning in Practice (LIP)
- ☐ Other: ……………………………………………………………………………………...................

Q11 Where do you record clinical information used for HANDOVER? (tick all that apply)

☐ Committed to memory	☐ paperPRF	☐ Back of ungloved hand
☐ Back of gloved hand	☐ ePRF	☐ Notes on scrap paper/notepad

☐ Other: ………………………………………………………………………………………..............................

Q12 Are you aware of any of the following HANDOVER mnemonics/formats? (tick all that you are aware of)

- ☐ **ASHICE** (Age, Sex, History, Injury/Illness, Condition, Expected time of arrival)
- ☐ **IMIST AMBO** (Identification, Mechanism/Medical Complaint, Injuries/Relevant info, signs, Treatment and Trends , Allergies, Medication, Background History, Other info)
- ☐ **SBAR** (Situation, Background, Assessment, Recommendations)
- ☐ **ATMIST** (Age/sex, Time of incident, Mechanism, Injuries found, Signs and Symptoms, Treatment provided, ETA)
- ☐ **MIST** (Mechanism, Injuries sustained or suspected, Signs – vital signs, Treatment)
- ☐ **De MIST** (Patient demographics, (Mechanism, Injuries sustained or suspected, Signs – vital signs, Treatment)
- ☐ **SOAP** (Subjective information, Objective information, Assessment, Plan)
- ☐ None/Other (if OTHER please specify):..……………………………………………….........................
……

Please turn over

Q13 Which, if any, of the following HANDOVER mnemonics/formats do you routinely use? (tick all that apply)

☐ **ASHICE** (Age, Sex, History, Injury/Illness, Condition, Expected time of arrival)

☐ **IMIST AMBO** (Identification, Mechanism/Medical Complaint, Injuries/Relevant info, signs, Treatment and Trends , Allergies, Medication, Background History, Other info)

☐ **SBAR** (Situation, Background, Assessment, Recommendations)

☐ **ATMIST** (Age/sex, Time of incident, Mechanism, Injuries found, Signs and Symptoms, Treatment provided, ETA)

☐ **MIST** (Mechanism, Injuries sustained or suspected, Signs – vital signs, Treatment)

☐ **De MIST** (Patient demographics, (Mechanism, Injuries sustained or suspected, Signs – vital signs, Treatment)

☐ **SOAP** (Subjective information, Objective information, Assessment, Plan)

☐ **None/Other** (if OTHER please specify):..

Q 14 If you had a choice of prehospital HANDOVER mnemonic which, if any, of the following would you choose (please tick only one)

☐ **ASHICE** (Age, Sex, History, Injury/Illness, Condition, Expected time of arrival)

☐ **IMIST AMBO** (Identification, Mechanism/Medical Complaint, Injuries/Relevant info, signs, Treatment and Trends , Allergies, Medication, Background History, Other info)

☐ **SBAR** (Situation, Background, Assessment, Recommendations)

☐ **ATMIST** (Age/sex, Time of incident, Mechanism, Injuries found, Signs and Symptoms, Treatment provided, ETA)

☐ **MIST** (Mechanism, Injuries sustained or suspected, Signs – vital signs, Treatment)

☐ **De MIST** (Patient demographics, (Mechanism, Injuries sustained or suspected, Signs – vital signs, Treatment)

☐ **SOAP** (Subjective information, Objective information, Assessment, Plan)

☐ **None/Other** (if OTHER please specify):..
....

Q15 In relation to your answer to Q14 (immediately above), do you feel your chosen HANDOVER mnemonic provides all the essential information

☐ Yes

☐ No (please state what you feel is missing in space provided below)

....
....

Q16 Do you feel the type of injury/illness (trauma/medical) influences your HANDOVER mnemonic of choice

☐ Yes (please state why in space provided below)

☐ No (please state why in space provided below)

....
....

Please turn over

Q17 Please read the following statements about HANDOVER in Glasgow Royal Infirmary resuscitation room, think of your practice and experiences, and rate your response on the scale provided (Never to Always)					
Statements	Never	Rarely	Sometimes	Often	Always
I feel I provide the information the ED staff need	☐	☐	☐	☐	☐
Is structured	☐	☐	☐	☐	☐
Is standardised (in the same format/same mnemonic)	☐	☐	☐	☐	☐
Is clear (volume)	☐	☐	☐	☐	☐
Avoids repetition	☐	☐	☐	☐	☐
Is listened to without interruption	☐	☐	☐	☐	☐
Hospital staff ask further questions	☐	☐	☐	☐	☐
Staff are ready and prepared	☐	☐	☐	☐	☐

This section is about pre-alert and handover aide-mémoire's

Q 18 Would you find an aide-mémoire useful in supporting pre-alert and handover?

☐ Yes (please provide details in space below)

☐ No (please provide details in space below)

……
……

Q 19 If you were to have an aide-mémoire, which of the following locations would you prefer to have access to this for pre-alert and handover? (tick all that apply)

Pre-alert	Handover	
☐	☐	Front cab of ambulance (paper based)
☐	☐	Patient treatment area of ambulance (paper based)
☐	☐	Pocket book (paper based)
☐	☐	ePRF system (front cab)
☐	☐	ePRF system (patient treatment area)
☐	☐	Other (if OTHER please specify): ………………………………………………………… …………………………………………………………

Please provide any additional comments with regards stand-by or handover here:

……
……
……
……
……

Thank you for taking the time to complete this survey. The results will help inform prehospital alert and handover practice within NHS Greater Glasgow and Clyde. Results will be published on station once analysis is completed.
